# Extensive thoracoabdominal aortic aneurysm as initial presentation in Takayasu arteritis: case series and literature review

**DOI:** 10.1093/ehjcr/ytad627

**Published:** 2023-12-18

**Authors:** Anupam Jena, Subasis Mishra, Binayananda Padhee, Surya Kant Jena, Nelson Ghosh, Prasanta Padhan, Nikunja Kishore Rout, Panchanan Sahoo

**Affiliations:** Department of Cardiology, Kalinga Institute of Medical Sciences, Campus 5, KIIT University, Patia, Bhubaneswar, India; Department of Cardiology, Kalinga Institute of Medical Sciences, Campus 5, KIIT University, Patia, Bhubaneswar, India; Department of Cardiology, Kalinga Institute of Medical Sciences, Campus 5, KIIT University, Patia, Bhubaneswar, India; Department of Cardiology, Kalinga Institute of Medical Sciences, Campus 5, KIIT University, Patia, Bhubaneswar, India; Department of Cardiology, Kalinga Institute of Medical Sciences, Campus 5, KIIT University, Patia, Bhubaneswar, India; Department of Immunology, Kalinga Institute of Medical Sciences, KIIT University, Patia, Bhubaneswar, India; Department of Nephrology, Kalinga Institute of Medical Sciences, KIIT University, Patia, Bhubaneswar, India; Department of Cardiology, Kalinga Institute of Medical Sciences, Campus 5, KIIT University, Patia, Bhubaneswar, India

**Keywords:** Takayasu’s arteritis, Aortic aneurysm, Endovascular repair, Thoracoabdominal aneurysm, Case series, Chimney EVAR

## Abstract

**Background:**

Aortic aneurysm as a presenting feature in Takayasu’s arteritis is very rare. Here, we report three cases of extensive thoracoabdominal aortic aneurysm in Takayasu’s arteritis as initial presentation.

**Case summary:**

All three cases were males and presented with complaints of abdominal pain and refractory hypertension. The diagnosis was made from the finding of thickened and calcified aortic wall, stenosis of visceral arteries, and age < 40 years at diagnosis. Case 1 was a 34 years male with aortic aneurysm extending from left subclavian artery to infrarenal aorta. He underwent endovascular repair of aneurysm by sandwich chimney technique in view of impending aneurysm rupture. Case 2, a 37 years male had aortic aneurysm from descending thoracic aorta (D4 vertebral body) to infrarenal aorta (L4 level). While being evaluated for repair, he had sudden death probably due to ruptured aneurysm. Case three, a 40 years male had aortic aneurysm extending from left subclavian artery to aortic bifurcation and stenosis of visceral arteries. He did not consent for repair and died one year later due to chronic kidney disease and related complications.

**Discussion:**

Thoracoabdominal aortic aneurysm is a very rare manifestation in Takayasu’s arteritis; more common in males. Endovascular repair is challenging but feasible. Long-term monitoring and repeat intervention may be needed due to young age of patients and disease progression.

**Learning points**

- Thoracoabdominal aortic aneurysm as a presenting feature in Takayasu’s arteritis is extremely rare.
- Endovascular repair by chimney technique is feasible in emergent situations; however, needs regular follow-up for disease progression and development of endoleaks.
- Untreated cases have worse prognosis.

## Introduction

Takayasu’s arteritis (TA) is characterized by stenotic lesions of the aorta and its major branches in >90% of cases.^1^ The reported incidence of aneurysms in TA is 25%.^1^ Thoracoabdominal aortic aneurysms (TAAAs) in TA are reported in 0.2% to 2% of cases.^2,3^ Here, we report three patients with TA whose initial presentation was with extensive TAAA (Type II by Crawford classification,^4^) out of whom one patient underwent endovascular repair.

## Summary figure

**Figure:**
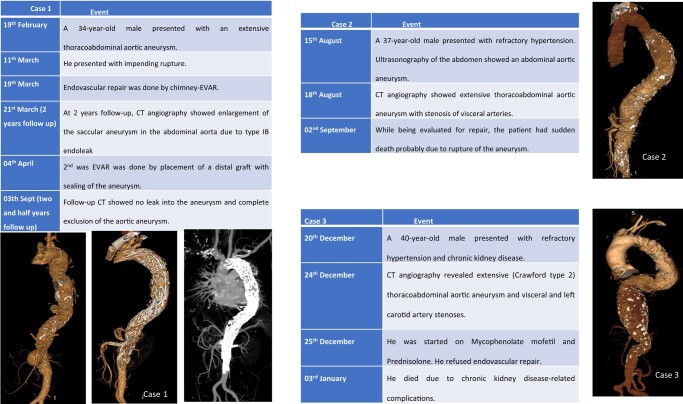


## Clinical summary

### Patient 1

**Figure 1:**
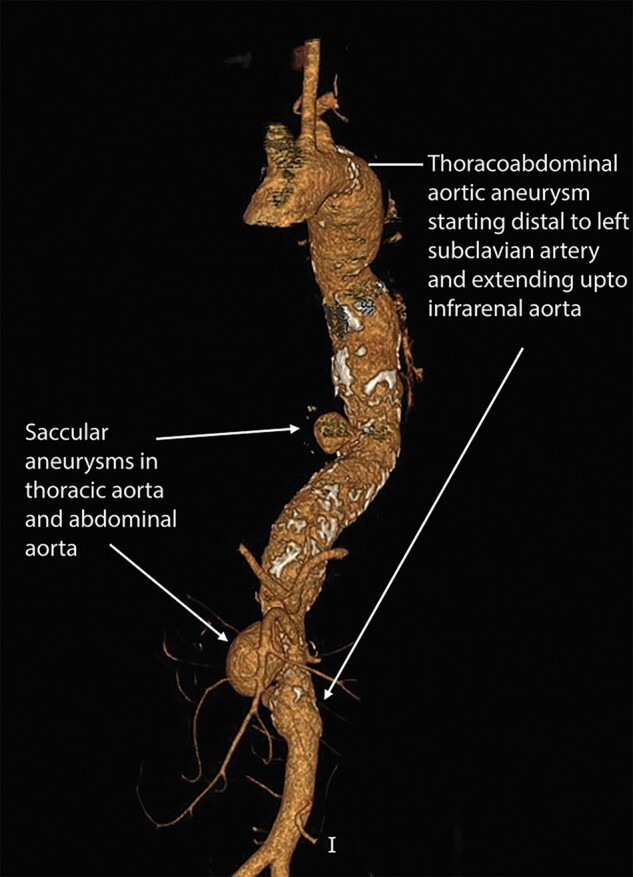
CT aortography. 3D reconstructed image showed extensive (type II) thoracoabdominal aortic aneurysm extending from the left subclavian artery to the infrarenal aorta. Aortic arch branches were normal.

A 34-year-old Asian male presented with complaints of abdominal pain; no previous comorbidity; blood pressure at presentation = 220/110 mmHg and pulse rate = 90/min; per abdominal, chest, cardiovascular and neurological examinations were unremarkable. Haemoglobin (Hb) = 9000, total leucocyte count (TLC) = 9000, C-reactive protein = 7.5 mg/dL, and erythrocyte sedimentation rate (ESR) was 50 mm/h. Abdominal ultrasound showed a shrunken right kidney and aneurysm of the abdominal aorta. Computerized tomography (CT) angiography of the aorta revealed a TAAA starting distal to the left subclavian artery and extending up to the infrarenal aorta with extensive wall calcification. The maximum diameter of the aneurysm in the descending thoracic aorta was 5.8 cm. There were two saccular aneurysms—one in the descending thoracic aorta measuring 3.4 cm × 3 cm and another in the abdominal aorta of size 6.2 cm × 4.2 cm (*Figure 1*). The right renal artery was completely occluded. Functional assessment of the right kidney by diethylenetriamine pentaacetate scan showed a severely reduced glomerular filtration rate of 5.62 mL/minute. The coeliac artery was severely stenosed at the ostium. Coronary angiography was normal. The Tuberculin skin test was negative, and contrast-enhanced CT of the abdomen and chest did not show evidence of tuberculosis. Evaluation for syphilis, mycotic aneurysms, Marfan syndrome, HLA-B27, antinuclear antibody, and blood cultures was negative. In view of age < 40 years, aortic aneurysm, complete occlusion of right renal artery, and stenosis of coeliac artery, the patient was diagnosed as a case of TA. Oral prednisolone 80 mg per day and mycophenolate mofetil 500 mg thrice daily (increased two weeks later to 1 g thrice daily) were started. As per the advice of clinical immunologists, the endovascular repair was planned after the control of disease activity. Three weeks later, the patient presented with complaints of severe pain in the abdomen. Computerized tomography angiogram of the aorta showed impending rupture of the saccular aneurysm in the abdominal aorta. Due to a life-threatening emergency, we decided to perform endovascular repair of the TAAA by sandwich chimney technique with chimney stents to the superior mesenteric artery (SMA) and the left renal artery. The coeliac artery was being supplied retrogradely by collaterals from the SMA. The right kidney was non-functional, so no revascularization was deemed necessary.

**Figure 2:**
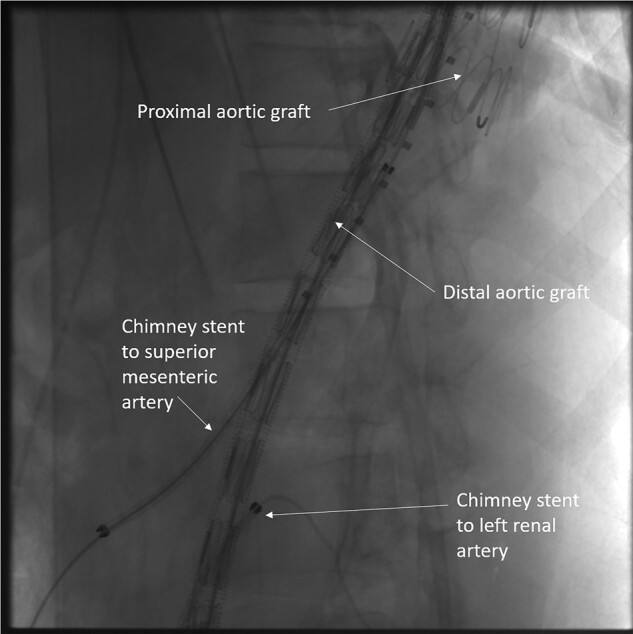
Endovascular aneurysm repair. The thoracic aortic stent graft was deployed first. The SMA stent was placed through the implanted graft. The stent in the left renal artery was placed from the left femoral artery, and the second aortic stent graft was positioned from the right femoral artery.

**Figure 3:**
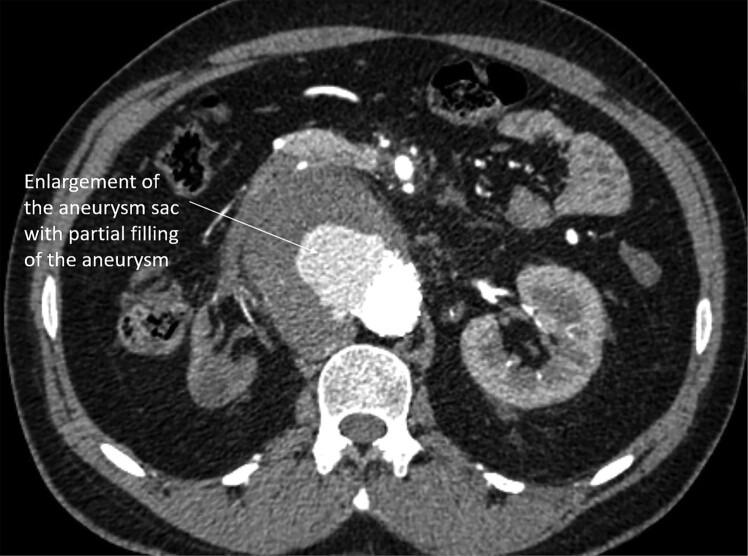
Follow-up CT. CT after two years showed contrast enhancement in the abdominal aortic saccular aneurysm and enlargement of the aneurysm.

**Figure 4:**
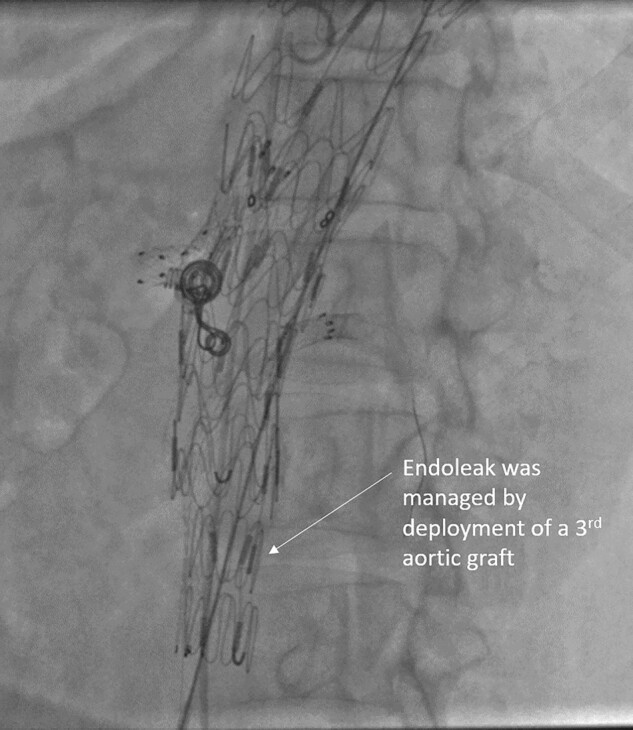
Endoleak repair. Type IB endoleak was treated by placement of a third aortic stent graft distally.

**Figure 5:**
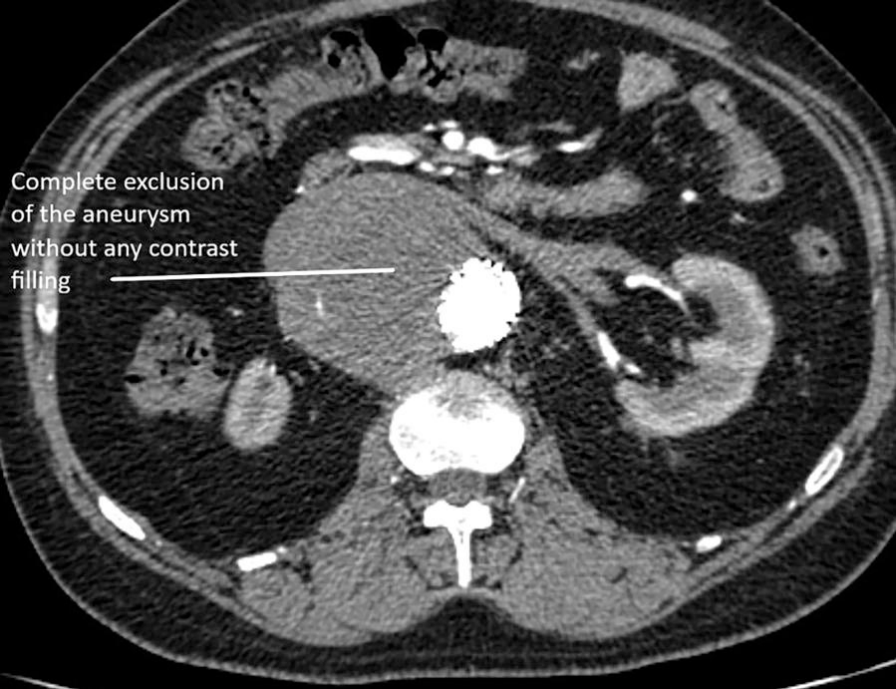
Follow-up CT. Complete obliteration of the saccular aneurysm in the abdominal aorta.

Endovascular repair: Vascular access was obtained from bilateral femoral and brachial arteries. Aortic stent graft 34 × 30 × 200 mm (Ankura™ TAA stent graft, Lifetech Scientific, Shenzhen, China) was deployed distal to the left subclavian artery. The SMA was stented with 8 × 100 mm and 10 × 60 mm covered stents (Fluency™ plus vascular stent, C.R. Bard Inc., USA). A second aortic stent graft of size 34 × 26 × 200 mm was deployed from the distal thoracic aorta to the infrarenal aorta. The first and second aortic graft overlap was 6 to 7 cm (*Figure 2*). The SMA stent was sandwiched between the two grafts. The left renal artery was stented with 6 × 80 mm covered stent (Fluency™ plus vascular stent, C.R. Bard Inc., USA) through the left femoral artery. No endoleaks were noted on the final angiogram. The coeliac artery was not stented as it was getting retrograde supply from SMA. The patient was followed up with CT aortography every six months. At two two-year follow-ups, CT-angiography revealed enlargement of the saccular aneurysm in the abdominal aorta due to type IB endoleak (*Figure 3*). The endoleak was due to the distal progression of the disease in the infrarenal aorta (see Supplementary material online, video  *S1*). It was managed by the deployment of 28 × 28 × 100 TAA Stent Graft (Ankura™ TAA stent graft, Lifetech Scientific, Shenzhen, China) distally (*Figure 4*, Supplementary material online, video  *S2*). The left renal stent was reinforced by deployment of a 6 ∗ 27 mm stent (Visi-pro™, Medtronic Inc., USA). The post-procedure aortogram confirmed the exclusion of the aneurysm and patent flow in SMA and left renal arteries. Follow-up CT shows complete exclusion of the aneurysmal segments (*Figure 5*). The patient is on 75 mg of aspirin daily, antihypertensives, oral prednisolone, and tofacitinib.

### Patient 2

**Figure 6:**
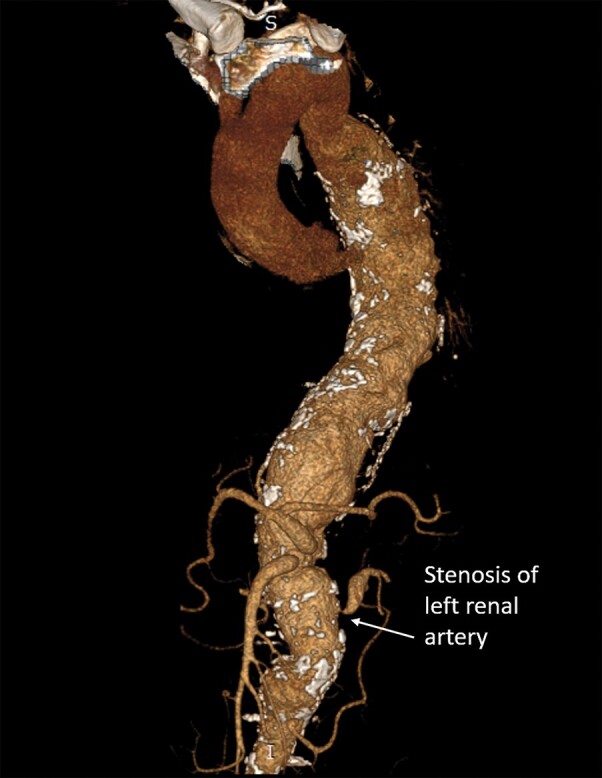
3D reconstructed images show a thoracoabdominal aortic aneurysm extending from the distal to the left subclavian artery up to the infrarenal aorta.

**Figure 7:**
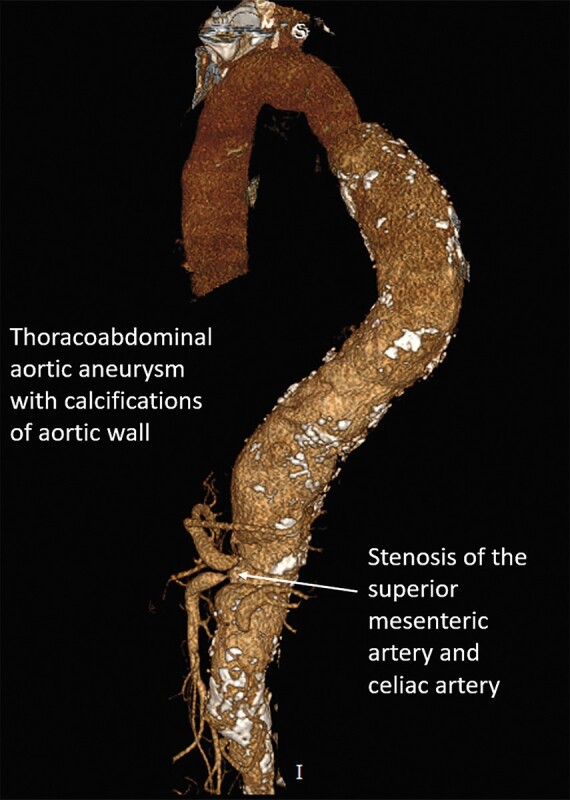
Stenosis of visceral arteries. Severe stenosis of the ostium of the superior mesenteric artery, coeliac artery, and left renal artery. The right renal artery was completely occluded.

A 37-year-old Asian male (no previous comorbidity) presented with refractory hypertension. Upon examination, pulse was 80/min, and blood pressure was 180/110 mmHg. Systemic examination was unremarkable. Erythrocyte sedimentation rate was 90 mm in 1st hour, and C-reactive protein was 7 mg/dL, Hb = 13, TLC = 13000, and normal renal parameters. Ultrasonography of the abdomen showed abdominal aortic aneurysm. Computerized tomography angiography revealed an aneurysm involving the descending thoracic and abdominal aorta extending from D4 (dorsal) to L4 (lumbar) vertebral bodies (*Figure 6*). The maximum diameter of the aneurysm in the descending thoracic aorta was 5.89 cm, and in the abdominal aorta, it was 5.57 cm. Severe stenoses were noted at the origin of the coeliac trunk, SMA, and bilateral renal arteries (right > left) (*Figure 7*). A diagnosis of TAAA due to TA was made in view of age < 40 years, involvement of thoracic and abdominal aorta, and ostial stenosis of visceral arteries. The Tuberculin skin test was non-reactive, and a CT scan of the chest and abdomen did not show evidence of tuberculosis. Evaluations for syphilis, mycotic aneurysm, atherosclerosis, and other connective tissue diseases were negative. While being evaluated for endovascular aneurysm repair, he had a sudden death, probably due to a ruptured aortic aneurysm.

### Patient 3

**Figure 8:**
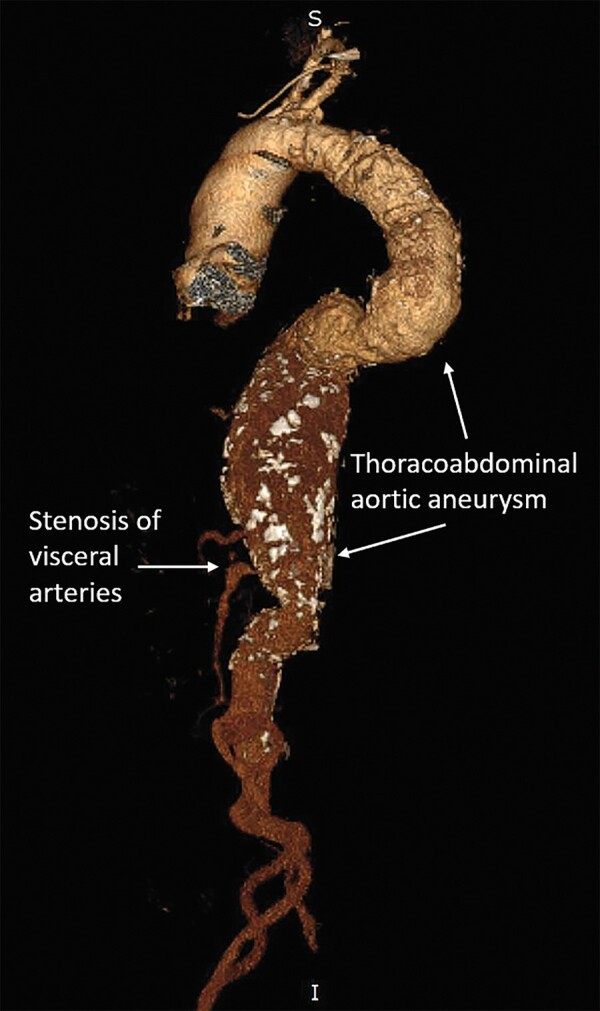
3D reconstructed images showing extensive thoracoabdominal aortic aneurysm extending from the left subclavian artery up to the aortic bifurcation. Stenosis of ostia of superior mesenteric, coeliac artery ostia, and left renal artery. The right renal artery was completely occluded.

A 40-year-old Asian male (no previous comorbidity) presented with uncontrolled hypertension and chronic kidney disease. Upon examination, the pulse rate was 90/min; the blood pressure was 200/100 mmHg in the right upper arm. On systemic examination, there was a 5 cm × 5 cm pulsatile mass above the umbilical level. Hb was 9.2, TLC = 7700, ESR was 110 mm in the 1st hour, C-reactive protein was 8.3 mg/dL, serum urea = 87 mg%, and creatinine = 2.8 mg%. An aortic aneurysm was detected on abdominal ultrasound. Computerized tomography aortography showed an aneurysm of the ascending aorta (maximum diameter 4.4 cm), descending thoracic and abdominal aorta extending from D2 vertebral body (distal to left subclavian artery) to L4 vertebral body (aneurysm size 7.03 cm). Severe ostial stenosis of the SMA, both renal arteries, coeliac artery, and left common carotid artery (*Figure 8*) was observed. In view of age (40 years), visceral artery stenosis, aortic aneurysm, and carotid stenosis, the patient was diagnosed as a case of TA. Evaluations for syphilis, mycotic aneurysm, atherosclerosis, and other connective tissue diseases were negative. There was no evidence of tuberculosis. The patient opted for conservative management. He was put on oral prednisolone, mycophenolate mofetil, and antihypertensives. The patient died after one year due to complications of chronic kidney disease.

## Discussion

Here, we present three male patients presenting with extensive TAAA due to TA. The diagnosis of TA was made in these three patients by the presence of the following Ishikawa criteria: (1) age < 40 years, (2) high ESR, (3) hypertension, (4) thoracic aortic involvement, and (5) abdominal aortic involvement.^5^ Published series from Mexico (10%), France (12.2%), Italy (7%), and India (9.1%) reported similar incidence of aneurysms in TA.^6^ In two large Japanese cohorts, the incidence of aneurysms was 32% and 15%, respectively.^3,7^ The most commonly reported site of aneurysm is ascending aorta (44%).^3^ Cheng *et al*.^6^ reported aneurysms in 16.6% of the patients with TA, with the most common site being the abdominal aorta (22%). Thoracoabdominal aortic aneurysms in Takayasu arteritis constituted 6% of all aneurysms in the series of Matsumura and co-workers (2% of the entire cohort) and 5% of the aneurysms in the series of Yang and associates (0.2% of the entire cohort).^2,3^ The most common symptoms were hypertension and abdominal pain.^8^ Current guidelines recommend repair of aneurysms at a diameter more than 5.5 mm or rapidly expanding aneurysms (>0.5 cm in six months or >1 cm per year), and for saccular aneurysms, early repair is recommended.^9^ According to Orr *et al*.,^10^ open surgical repair of TAAA results in significantly more morbidity than endovascular repair.

Endovascular techniques for aneurysm repair and preservation of visceral artery patency include custom-made fenestrated endovascular aneurysm repair (f-EVAR), branched endovascular aneurysm repair (b-EVAR), and chimney EVAR. Because of the limited availability of fenestrated and branched endografts (f-EVAR and b-TEVAR) and for the long time needed for customization (up to eight weeks), parallel grafting is being increasingly used in urgent and emergent settings.^11^ The chimney technique (Ch-EVAR) involves the placement of a stent graft parallel to the main aortic stent graft. The chimney stent maintains patency of the side branch and extends the proximal or distal sealing zones. The concerns with Ch-EVAR are endoleaks, bridging stent patency, and long-term outcomes.^12^ However, early and midterm outcomes seem to have no significant differences between Ch-EVAR, f-EVAR, and b-EVAR techniques.^12,13^ Pre-operative CT helps with the evaluation of (1) the diameters of proximal and distal aortic landing zones, (2) the number and diameters of branch vessels to be grafted, and (3) vascular access. Most of endovascular strategies need upper arm brachial access; hence, understanding arch anatomy to ensure the safe passage of sheaths and wires without increasing the risk of cerebrovascular events is vitally important. The aortic stent graft should be oversized by 30% in relation to the aortic landing zone.^11^ We opted for Ch-EVAR in this case due to tortuous aorta, presentation with impending aneurysm rupture warranting early aortic repair. The Ch-EVAR technique ensures immediate exclusion of the aortic aneurysm and maintenance of patency of the renal and superior mesenteric arteries.^14,15^ In our case, self-expandable chimney grafts were used. The graft overlap was >60 mm for the SMA graft and the second aortic graft. For the renal artery graft, the overlap was 50 mm. Lobato *et al*. reported 98.7% technical success of the sandwich technique. Over a mean 17-month follow-up (range 1–42), primary patency was high (96.7%), mortality low (early: 5.1%, late: 1.3%), and persistent endoleak in 5.1% cases.^16^ We could achieve complete exclusion of the TAAA with patent chimney grafts without type I or III endoleak. Problems specifically associated with Ch-EVAR include the necessity for upper limb arterial access, which can result in ischaemic stroke in 3.2% of cases, chimney stent graft compression, and gutter endoleak.^17^ In elective cases, fenestrated and branched aortic grafts can be used, which were custom-made to fit the patient’s anatomy.^18^

At two years of follow-up, persistent flow into the saccular aneurysm in the abdominal aorta was noted. The possibility of a gutter leak was evaluated.^19^ However, the aortogram was suggestive of type IB endoleak due to progression of the disease distally, resulting in loss of sealing at the distal end of the graft. As type 1 endoleak develops, the renewed pressure on the weakened aortic wall has an increased rupture risk in comparison to an unrepaired aneurysm with the same diameter. Therefore, both proximal and distal type 1 endoleaks need reintervention.^20,21^ Studies have documented an increase in the aortic diameter at the transition zone from the stent graft into the non-stented aorta. The spiralling bloodstream enters the stent graft and has to change its original vortex because of a significant change in shear stresses inside a long segmental stent graft compared with the elastic properties of a native non-stented aorta. This might be responsible for an energy burst at the outlet of the stent graft, inducing dilatation of the native aorta at this transition zone.^22^ Our case was managed by placement of a third graft distally that excluded the aneurysm.

## Conclusion

Thoracoabdominal aortic aneurysms are rare in TA, with a higher incidence in males. The presence of TAAA significantly increases the complications of TA, like chronic kidney disease, aortic rupture, and dissection. Total endovascular repair, though challenging, is feasible. Regular follow-up is necessary with periodic CT aortograms. There may be a need for repeat interventions on long-term follow-up.

## Supplementary Material

ytad627_Supplementary_Data

## Data Availability

The data underlying this article will be shared on reasonable request to the corresponding author.

## Lead author biography

Dr Anupam Jena is an Assistant Professor in the Department of Cardiology at the Kalinga Institute of Medical Sciences, Bhubaneswar, India. He is engaged in clinical work in cardiology for the last 12 years. He is an interventional cardiologist and has expertise in endovascular aortic repair and interventions for peripheral arterial diseases. He has proficiency in management of complex coronary interventions like CTO, left main intervention, and etc. He is the pioneer of interventions for structural heart disease in Odisha state.

## Supplementary material

**Consent:** The authors confirm that written consent for submission and publication of this case report, including images and associated text, has been obtained from the patient in line with COPE guidance.

**Funding:** None.
