# Supplementary figures and images for: Extensive thoracoabdominal aortic aneurysm as initial presentation in Takayasu arteritis: case series and literature review
Source: Eur Heart J Case Rep. 2023 Dec 18;8(1):ytad627. doi: 10.1093/ehjcr/ytad627 (PMC10766067; doi:10.1093/ehjcr/ytad627)

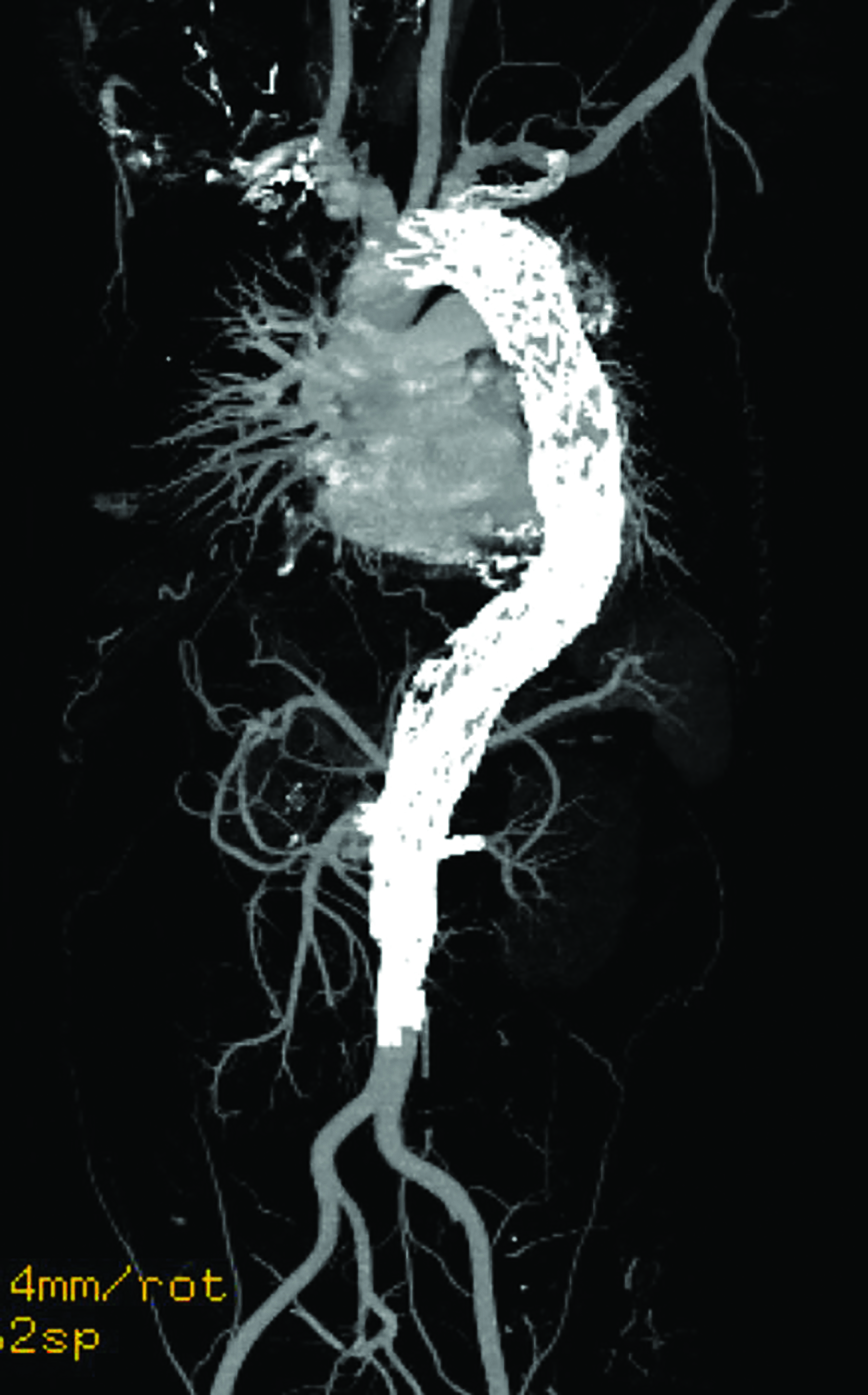

Supplement: ytad627_Supplementary_Data [file ytad627_supplementary_data.zip › Supplementary Figure 1.JPG]
